# Response to comment on ‘SARS-CoV-2 suppresses anticoagulant and fibrinolytic gene expression in the lung’

**DOI:** 10.7554/eLife.74951

**Published:** 2022-01-11

**Authors:** Alan E Mast, Alisa S Wolberg, David Gailani, Michael R Garvin, Christiane Alvarez, J Izaak Miller, Piet Jones, Bruce Aronow, Daniel Jacobson

**Affiliations:** 1 Versiti Blood Research Institute, Department of Cell Biology Neurobiology and Anatomy Medical College of Wisconsin Milwaukee United States; 2 Department of Pathology and Laboratory Medicine and UNC Blood Research Center Chapel Hill United States; 3 Department of Pathology, Microbiology and Immunology, Vanderbilt University Medical Center Nashville United States; 4 Oak Ridge National Laboratory, Biosciences Division Oak Ridge United States; 5 University of Tennessee Knoxville, The Bredesen Center for Interdisciplinary Research and Graduate Education Knoxville United States; 6 Biomedical Informatics, Cincinnati Children’s Hospital Research Foundation, University of Cincinnati Cincinnati United States; Kobe Pharmaceutical University Japan; Radboud University Medical Centre Netherlands

**Keywords:** COVID-19, coagulation, fibrinolysis, bronchoalvelolar, SARS-CoV-2, Human

## Abstract

Early in the SARS-CoV-2 pandemic, we compared transcriptome data from hospitalized COVID-19 patients and control patients without COVID-19. We found changes in procoagulant and fibrinolytic gene expression in the lungs of COVID-19 patients (Mast et al., 2021). These findings have been challenged based on issues with the samples (Fitzgerald and Jamieson, 2022). We have revisited our previous analyses in the light of this challenge and find that these new analyses support our original conclusions.

## Introduction

As part of the global effort to address the pandemic caused by the SARS-CoV-2 virus, we support a robust discussion on COVID-19 to provide insight into the biological effect of the virus on humans. Previously, we compared transcriptome data from hospitalized COVID-19 patients to control patients without COVID-19 and found changes in procoagulant and fibrinolytic gene expression in the lungs of COVID-19 patients (Mast et al., 2021). These findings were subsequently challenged based on issues with the samples, including library depth, library preparation, and control group meta-data (FitzGerald and Jamieson, 2022). We have revisited our previous analyses of these two data sets, and we find that they are comparable to one another and support our conclusions. We address each of the criticisms of FitzGerald and Jamieson, 2022 below.

## Results

### The designation of Michalovich et al. as a “Healthy Control” for differential expression analysis

Fitzgerald and Jamieson have criticized our results based on the fact that the control group is (1) not free of comorbidities, and (2) "not representative of the American population". In accordance with *eLife’s* transparent reporting procedures, we list group allocation as “Cases were those individuals diagnosed with COVID-19, controls were from a separate study in which participants were not diagnosed with any viral infection”. Our goal of the analysis was to test for differences in the expression of genes in the coagulation pathway in response to infection from the SARS-CoV-2 virus. Most of the BALF samples in Michalovich et al. harbored comorbidities such as asthma, nicotine dependence, and obesity. Of the 40 control samples, three were reported to be free of these comorbidities (putatively 'healthy'). We find the criticisms raised are not warranted for the following reasons:

1. Fitzgerald and Jamieson make the assumption that the patients displaying COVID-19 from which the BALF samples were taken were healthy and had no comorbidities, however, comorbidities increase the risk of hospitalization with severe disease as well as mortality (King et al., 2020). We made no assumptions about the case samples and used what we believe to be a conservative approach by using all 40 control samples. Statistical significance may be more difficult to achieve with greater variance, therefore, only the strongest signals due to infection of the virus would be discovered.
2. Sub-group analysis can be informative but is vulnerable to Simpson’s paradox, ascertainment bias, and statistical power variability (unequal representation for each sub category). We did not perform a sub-group analysis as this was beyond the scope of the paper and not related to the hypothesis, which as we note above was differences in gene expression due to infection with a virus.
3. The samples of Michalovich et al. are from Poland and Switzerland, not the United States of America.

### Dissimilar library preparation methods of Michalovich et al. (transcriptomic) and Zhou et al. (total RNA) are not comparable

**Figure 1.**
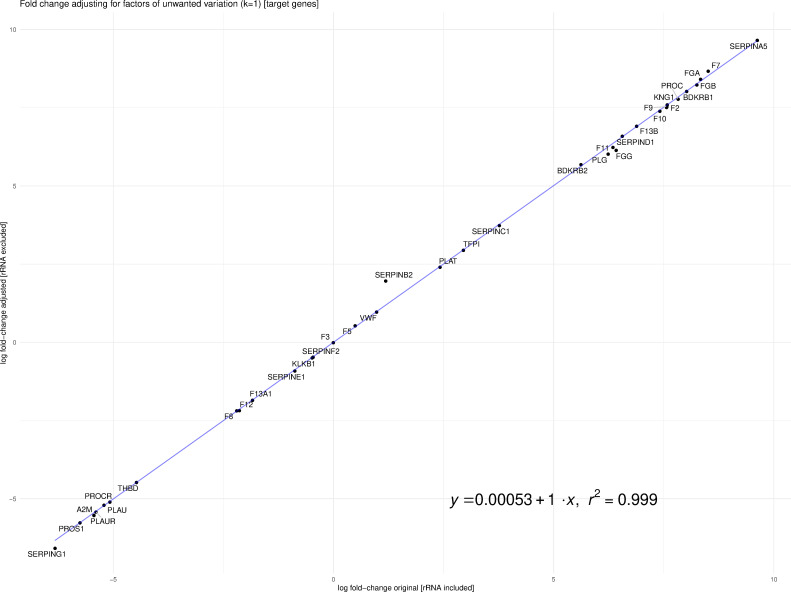
Correlation between log-fold change with rRNA and the adjusted log-fold change without rRNA (R2 ~0.99954).

Fitzgerald and Jamieson state that dissimilar library preparation methods of Michalovich et al. (Transcriptomic) and Zhou et al. (Total RNA) are not comparable. Library preparation for the case and control samples was different for the two studies. Michalovich et al. used a polyA enrichment method, which removes most ribosomal RNA (rRNA), and for the 9 cases, total RNA that includes rRNA was prepared. However, the assumption that this makes the samples non-comparable is flawed. Previous work in the field of RNA-Seq analysis has developed tools and techniques to mitigate sources of technical variation, such as library preparation (Risso et al., 2014). Our analysis leveraged TMM normalized counts for the differential analysis, implemented in edgeR. Fitzgerald and Jamieson raise concerns about our differential analysis based on the findings of Zhao et al., 2020. However, Zhao et al. focused on TPM and did not discuss more robust normalization protocols such as TMM or RLE, and thus may not be applicable in this case. To address the concerns of Fitzgerald and Jamieson, we re-calculated log-fold change of the differentially expressed genes from our analysis after removing rRNA counts from the CLC Genomics output. Furthermore, we accounted for factors (k = 1) of putative technical variation using the RUVSeq R package thereby producing an adjusted log-fold change (Risso et al., 2014). As one can see in Figure 1, the log-fold change with rRNA and the adjusted log-fold change without rRNA are highly correlated (R^2^ ~0.99954).

In addition, keeping all non-COVID-19 controls helps to resolve the issue where a gene is observed in COVID-19 samples but has few reads in the controls.

### Insufficient read depth of samples from Zhou et al.

**Figure 2.**
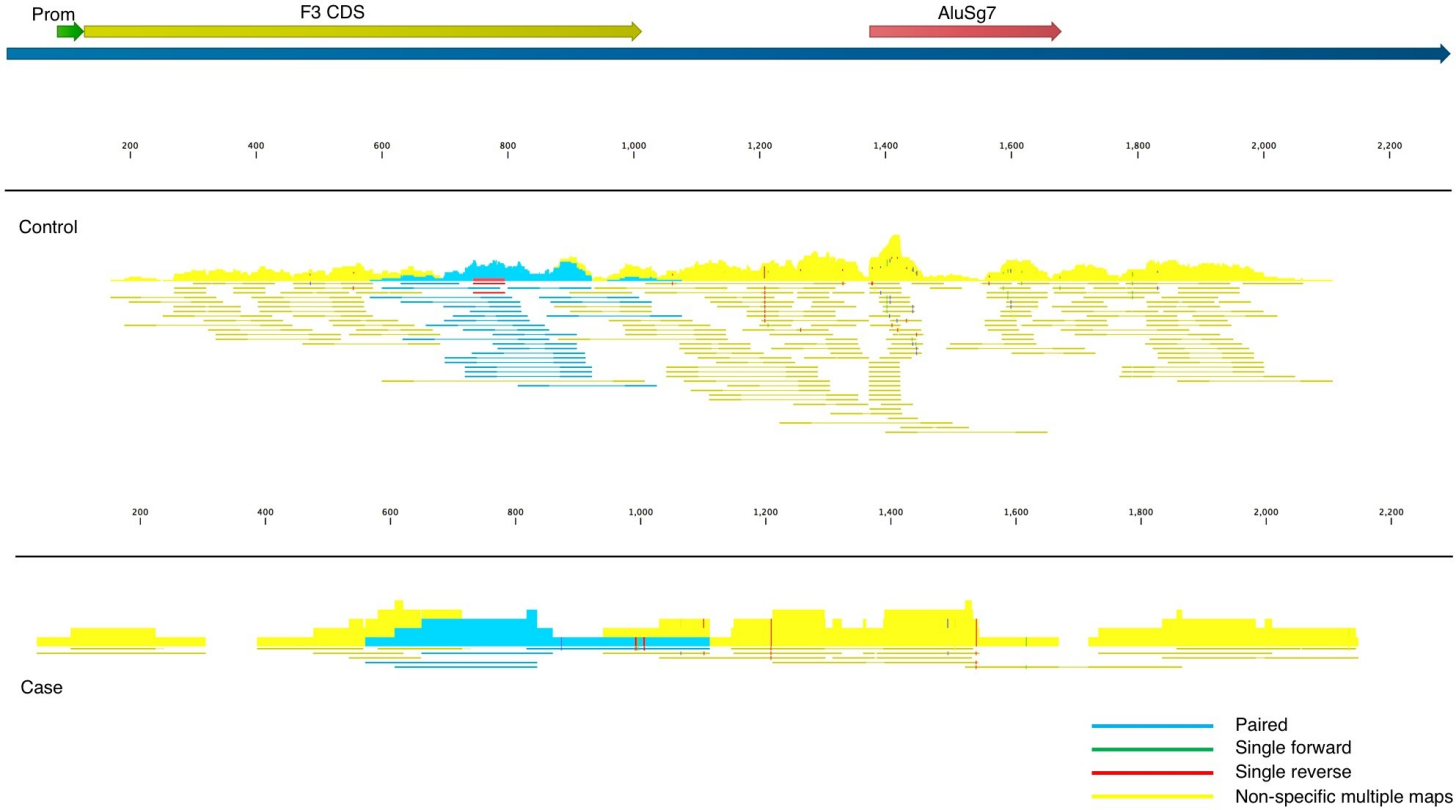
Read mappings to the transcript NM_001993 for the F3 mRNA taken from one of the controls (top) and a case (bottom) used in Mast et al. Mapping settings were set to mismatch cost = 2, insertion cost = 3, deletion cost = 3, length fraction = 0.95, similarity fraction = 0.95. The Alu element in the 3’UTR (brick), promoter (green), and coding sequence (dark yellow) of the transcript are annotated. Blue read mappings are paired reads that mapped a single time and light yellow are read mappings that aligned to multiple places in the transcriptome (i.e. other genes besides F3).

Fitzgerald and Jamieson state that COVID-19 BALF samples contain insufficient read depth. We acknowledge that there exist “generally accepted rules” for read depth, etc. in the field of transcriptome analysis, and these guidelines are important to adhere to wherever possible. The nine BALF samples used in our analysis were taken from severely ill patients in Wuhan China in an attempt to identify the as-yet-unidentified pathogen causing their symptoms and were not prepared in conjunction with control samples. The low read-depth of the nine BALF samples resulted in high false-positive (off-target) counts due to the presence of *Alu* elements in some of the transcripts, which can be seen in the read mapping files (Figure 2). To account for this, and to reduce inflation or deflation of expression levels, we used highly stringent mapping parameters in CLC Genomics Workbench. The default parameters for this algorithm are to score a “match” for a read if at least 0.80 of the fragment matches the gene and if the similarity is 0.8 or greater. Instead, to reduce off-target mapping, we used the much more conservative approach of 0.95 length and 0.95 similarity match.

Given these highly stringent parameters, and the much larger read depth of the control samples, any genes that are expressed in COVID-19 BALF samples, but are not present in controls, have a high probability of being true positives and the fold-increase is likely an underestimate. We report large increases in many cases because the denominator of the controls was zero and therefore fold-change would be infinite. To account for this, we used the lowest number in the matrix of gene counts as the denominator.

## Discussion

Fitzgerald and Jamieson state “By far the most notable result reported in Mast et al. is the reported observation that tissue factor, the key initiator of the extrinsic coagulation cascade, is not significantly impacted by SARS-CoV-2 infection”. As noted previously, the presence of *Alu* elements can provide spurious read mappings in the situation where there are low read counts, as is the case here. Indeed, not only does tissue factor (*F3*) contain an *Alu* element in the 3’UTR of the gene, the read mapping file in CLC Genomics Workbench clearly shows for both cases and controls, that the majority of the fragments map to multiple places in the transcriptome (yellow in Figure 2), and cannot be assigned to *F3* with confidence. Regardless, in these BALF samples, *F3* does not appear to be expressed at the mRNA level in cases or controls to any appreciable level. *F3* may be upregulated in other tissues or cell types, or it may also be regulated at the protein and/or activity level, i.e., our analysis does not preclude a role of *F3* in the complex pathology of COVID-19. For example, increased tissue factor has been detected by flow cytometry in monocytes and platelet-monocyte aggregates (Hottz et al., 2020) and by confocal microscopy and RT-qPCR in neutrophils (Skendros et al., 2020) from critically ill COVID-19 patients. Interestingly, it has been reported that a SARS-CoV-2 spike protein pseudovirus increases tissue factor activity in cells by converting it from an inactive to active form without altering protein expression (“decryption”) (Wang et al., 2021), which is consistent with our results.

In their Discussion the authors state that “the field has begun converging on tissue factor as a key player in the pathogenesis and coagulopathy complications of SARS-CoV-2 infection”. As stated above, we applaud the scientific community for their continued focus on this pathway and we are proud to have contributed to it early in the pandemic. We trust that the community does not think that we claim to have provided a complete and final understanding of COVID-19’s effects in the lungs. Solutions to complex problems are achieved from a consensus of researchers addressing them with multiple approaches, one of which is our study.

## Data Availability

Previously published data sets with BioProject IDs PRJNA605983 and PRJNA434133 were used. The following previously published datasets were used: ZhouP YangXL WangXG HuB ZhangL ZhangW SiHR ZhuY LiB HuangCL ChenHD ChenJ LuoY GuoH JiangRD LiuMQ ChenY ShenXR WangX ZhengXS ZhaoK ChenQJ DengF LiuLL YanB ZhanFX WangYY XiaoGF ShiZL 2020Severe acute respiratory syndrome coronavirus 2 Raw sequence readsNCBI BioProjectPRJNA605983 SeshadriR MyersGS TettelinH EisenJA HeidelbergJF DodsonRJ DavidsenTM DeBoyRT FoutsDE HaftDH SelengutJ RenQ BrinkacLM MadupuR KolonayJ DurkinSA DaughertySC ShettyJ ShvartsbeynA GebregeorgisE GeerK TsegayeG MalekJ AyodejiB ShatsmanS McLeodMP SmajsD HowellJK PalS AminA VashisthP McNeillTZ XiangQ SodergrenE BacaE WeinstockGM NorrisSJ FraserCM PaulsenIT 2018Microbiome and Inflammatory Interactions in Obese and Severe Asthmatic AdultsNCBI BioProjectPRJNA434133
